# Does endovascular duration impact clinical outcomes in aortic arch repair? The RELAY™ branched international stance

**DOI:** 10.3389/fcvm.2022.969858

**Published:** 2022-07-18

**Authors:** Sven Z. C. P. Tan, Abedalaziz O. Surkhi, Matti Jubouri, Damian M. Bailey, Ian M. Williams, Mohamad Bashir

**Affiliations:** ^1^Barts and The London School of Medicine and Dentistry, Queen Mary University of London, London, United Kingdom; ^2^Faculty of Medicine, Al-Quds University, Jerusalem, Palestine; ^3^Hull-York Medical School, University of York, York, United Kingdom; ^4^Neurovascular Research Laboratory, Faculty of Life Sciences and Education, University of South Wales, Wales, United Kingdom; ^5^Department of Vascular Surgery, University Hospital of Wales, Wales, United Kingdom; ^6^Vascular and Endovascular Surgery, Velindre University NHS Trust, Health Education and Improvement Wales, Wales, United Kingdom

**Keywords:** thoracic aortic aneurysm, aneurysm, RELAY™, TEVAR, branched RELAY, custom-made device technology

## Abstract

**Background:**

The high mortality and morbidity rates in surgical aortic arch repair are a barrier to therapy for a considerable proportion of patients with aortic arch aneurysm or dissection. There is hence a demand for the development and adoption of a minimally invasive alternative to aortic arch repair, such as thoracic endovascular aortic repair (TEVAR). Procedural duration is a key factor in the pathogenesis of complications in surgical aortic arch repair. Herein, we evaluate whether endovascular duration impacts neurological outcomes, target vessel patency, and reintervention rates in aortic arch TEVAR with RELAY™ Branched (Terumo Aortic, Inchinnan, UK), which is specifically developed for on-label use within the aortic arch.

**Methods:**

Prospective data was collected between January 2019 and January 2022 on the clinical outcomes of TEVAR for aortic arch dissection and aneurysm with RELAY™ single-, double-, and triple branched endoprostheses from centers across Europe. They were then retrospectively analyzed with descriptive and distributive analysis. Follow-up data on the incidence of disabling stroke (DS), target vessel patency, and reintervention from 30 days and 6-, 12-, and 24 months postoperative was included in the analysis.

**Results:**

147 (99.3%) of all 148 cases were successful. Over the 24 month follow-up period, in total 6 (4.1%) patients suffered DS, 24 (16.3%) required reintervention, and target vessel patency was exhibited in 118 (80.2%) patients. The modal endovascular duration was 100–150 min (in 64.6%, *n* = 95 cases). Analysis revealed that endovascular duration was associated with a lower likelihood of reintervention at 30 days, 6-, and 12 months (*P* = 0.011, *P* = 0.019, *P* = 0.037), a greater likelihood of target vessel patency at 6- and 24 months (*P* = 0.032, *P* = 0.035). No relationship between endovascular duration and DS was revealed.

**Discussion:**

The data demonstrates that RELAY™ Branched is associated with promising clinical outcomes for on-label aortic arch TEVAR. The underlying mechanism linking endovascular duration and reintervention rates, or target vessel patency is likely multifactorial and complex. Given that TEVAR is carried out under general anesthetic only, it is unlikely that prolonged procedural duration has any major effect over neurological outcomes for arch TEVAR.

## Background

Surgical innovations such as endovascular aneurysm repair (EVAR) and thoracic endovascular aortic repair (TEVAR) have revolutionized the management of life-threatening aortic pathologies by providing a minimally-invasive alternative to open surgical repair (OSR). However, OSR remains the mainstay treatment for aortic arch aneurysm and dissection. Despite its widespread application and impressive success rates, OSR continues to be associated with an exceptionally high rates of mortality and morbidity; mortality rates as high as 32.0% have been reported for the surgical repair of complicated type B aortic dissection, while ~15.9% of patients undergoing open proximal aortic repair suffer permanent neurological complications (1). These statistics are especially striking when compared to those associated with TEVAR for type B aortic dissection. Furthermore, given the invasiveness of OSR for the aortic arch, which also requires cardiopulmonary bypass (CPB) and hypothermic circulatory arrest (HCA), the turn-down rate for open surgical arch reconstruction remains at an all-time high of up to 40% (2).

Clearly, given the risks associated with open surgical arch reconstruction, and the consequent limitations for patient eligibility, a similarly minimally invasive approach to aortic arch repair would be valuable. TEVAR, having been adopted as the mainstay approach to repair of the thoracic aorta, has been shown to be a promising alternative to OSR that negates the use of CPB, HCA, and aortic cross-clamping while facilitating deployment of custom-made endoprostheses to preserve native aortic stability while inducing false lumen (FL) obliteration or aneurysmal shrinkage (3).

As aortic arch TEVAR has increased in popularity, the challenges of endovascular navigation around the curvature of the aortic arch, avoiding iatrogenic coverage of the supra-aortic branches, preserving target vessel patency, and reducing reintervention rates have come to the fore (2). These have been met with the development of specialized custom-made branched and fenestrated endoprostheses. RELAY™ Branched (Terumo Aortic, Scotland), for example, is designed specifically for on-label deployment throughout the aortic arch, and is available in single-, double-, or triple-branched configurations.

Of particular concern during proximal aortic repair is the effect that procedural duration may exert on the pathogenesis of neurological and structural complications, namely perioperative disabling stroke (DS), loss of target vessel patency, and the need for reintervention. This effect has been investigated at length in the context of proximal aortic OSR, though evidence remains mixed (4). Yet, since the advent of aortic arch TEVAR, there has hitherto been no published investigation into the role played by procedural duration on the pathogenesis of these key complications.

In light of these findings, the present study was designed to explore the potential relationships between procedural duration and the pathogenesis of DS, reintervention rates, and target vessel patency in aortic arch TEVAR with RELAY™ Branched. We present a unique, multi-center analysis of prospective data from European centers, and provide unique insight into clinical outcomes.

### TEVAR for aortic arch pathology: Challenges and strategies

Unlike EVAR for thoracoabdominal aortic pathology, or TEVAR for thoracic aneurysm or dissection, endovascular torque control may be severely limited by the anteroposterior and mediolateral curvature of the proximal thoracic aorta (5). This makes accurate placement and deployment of the endoprosthesis challenging and may necessitate the use of buddy wires or through-and-through catheterization to improve operator control (5). Introduction of a guidewire through the femoral vein, advancing it through the inferior vena cava, crossing into the left heart *via* the atrial septum, and subsequently into the proximal aorta and out *via* the femoral artery (i.e., the transeptal approach) has been suggested to confer greater torque control than simple direct retrograde catheterization of the proximal aorta *via* through-and-through manipulation (5). The transeptal approach has also been suggested to provide improved access to zone 0 compared to the transbrachiofemoral approach as it avoids the sharp turn at the innominate artery (IA) ostium (5). Notably, the RELAY™ Branched delivery system features a pre-curved inner catheter and dual sheath to improve alignment with the aortic arch. The cannulation window situated on the dorsal aspect (from which the supra-aortic branches are cannulated) has radiopaque markers to aid device positioning relative to the arch branch ostia.

Care must also be taken when selecting the proximal landing zone for endoprosthesis deployment. Preoperative transoesophageal echocardiography (TOE) is typically employed to measure the distance from the coronary ostia and sinotubular junction to the proximal entry tear (in the case of proximal aortic dissection) (2). Endovascular repair may be contraindicated in patients with a primary entry tear within 20 mm of the sinotubular junction to avoid compromising coronary supply, and in patients with a zone 0 aortic diameter >38 mm (2, 6).

It should be also highlighted that planting the endoprosthesis deep within zone 0 exposes it to maximal hemodynamic pressure, increasing the risk of malorientation as a result of the windsock effect (7). In view of these spatial constraints, shorter and wider endoprosthetic dimensions are favored, and 15% oversizing of the endoprosthesis relative to the native aortic diameter is considered to improve sealing (8). This clearly justifies the need for custom-made aortic arch endoprostheses licensed for on-label use throughout zones 0–4, to which RELAY™ Branched is eminently suited. Notably, most TEVAR endoprosthesis are limited to on-label use in the descending thoracic aorta.

Though TEVAR of the aortic arch does not require HCA or CPB, the risk of cerebrovascular accident remains omnipresent. In the absence of such invasive anesthetic techniques, DS typically arises from inadvertent embolization of luminal plaques by endovascular instrumentation or inadvertent iatrogenic occlusion of the supra-aortic branches (9, 10). The risk of occlusive ischemic stroke is thought to be greater in patients undergoing TEVAR with planned occlusion of the left subclavian artery (LSA) and in those with a proximal landing zone in close proximity to the sinotubular junction (11). Maintenance of LSA patency circumvents the risk of inadequate collateralization leading to left arm ischemia, avoids subclavian steal syndrome (and resultant vertebrobasilar insufficiency), and has also been shown to carry a lower risk of stroke (12). Bradshaw and colleagues reported a 1.9% stroke rate in patients who underwent endovascular or extraanatomical LSA revascularization, in comparison to a 14.3% stroke rate in those that underwent TEVAR with total LSA occlusion (12). The triple-branched RELAY™ endoprosthesis allows cannulation of all three supra-aortic branches, and in patients where LSA cannulation is not feasible, extra-anatomical bypass (such as carotid-subclavian) can be performed prior to TEVAR with a double- or single-branched RELAY™ (13). The effect that increased procedural duration in cases involving triple-branched RELAY™ endoprosthesis may have on the risk of causing perioperative DS remains unclear.

Target vessel patency is a key clinical evaluative metric, and maintenance thereof is pivotal to disease regression, patient quality of life, and event-free survival. Loss of aortic or arch branch patency may potentiate reintervention, which further exposes the patient to perioperative risks and impacts quality of life. Inadequate proximal sealing and intimal injury increase the likelihood of postoperative endoleak, which remains Achilles heel of endovascular repair (14, 15). This emphasizes the importance of custom-made, appropriately sized endoprostheses for long-term durability. Furthermore, retrograde dissection of extension of dissection involving the supra-aortic branches compromises vessel patency, though it is reasonable to suggest that endovascular cannulation of the arch vessels may attenuate this effect (15). As a custom-made endoprosthesis designed for on-label use throughout zones 0–4 of the aortic arch, RELAY™ Branched can be designed in a bespoke manner to fit well with each patient's unique anatomy. Its availability in single-, double-, or triple-branched configurations allows further flexibility in maintaining patency of both the supra-aortic branches and the aortic arch. The built-in cannulation branches avoids the need for the chimney technique, which has been associated with an increased risk of endoleak and subsequent loss of aortic patency (the risk of which presumably increases the more proximally the endoprosthesis is positioned, owing to the windsock effect) (16).

## Methods

### Study design

A 24-month international multi-center retrospective analysis of key outcomes (DS, reintervention, and target vessel patency) in patients treated for aortic arch pathology using the RELAY™ Branched endoprosthesis was carried out between January 2019 and January 2022. Data were collected in a prospective fashion from European centers and stored in a registered database. IRB ethical review and approval was not required for the present study with human participants, in accordance with local legislation and institutional requirements.

### Patient characteristics

In total, 148 patients underwent endovascular repair of the aortic arch with the RELAY™ Branched endoprosthesis. Of these, 38 patients were female, resulting in a 3:1 male-female ratio. The mean age was 70 (IQR = 14.5) years. 72.3% (*n* = 107) of patients were treated for a proximal aortic aneurysm while 27.7% (*n* = 41) were treated for aortic dissection involving the aortic arch. 46% (*n* = 68) of patients were acute cases while 54% (*n* = 80) underwent elective endovascular repair. Patient characteristics are summarized in Table 1.

**Table 1:** Demographics.

Gender (male : female)		3:1
Mean age (IQR)		70 (14.5)
**Pathology (%)**		
Aneurysm	107 (72.3%)	
Dissection	41 (27.7%)	
**Urgency (%)**		
Acute	68 (46%)	
Elective	80 (54%)	
Regional distribution*		
West Europe	78 (52.7%)	
East Europe	32 (21.6%)	
North Europe	9 (6.1%)	
South Europe	29 (19.6%)	
Total cases		148

*Based on UNSD Geoscheme.

### Follow-up

Patients were followed-up at 30-days and 6-, 12-, and 24-months postoperatively. During follow-up, patients were evaluated for DS and target vessel patency, and all cases requiring reintervention during these intervals were recorded. DS was defined in accordance with the VARC-2 criteria which categorizes DS as a modified Ranking score (mRS) > 3, reflecting that the patient has moderate disability; requiring some external help but able to walk without the assistance of another individual. Cumulative mortality was also recorded at each follow-up interval.

### Statistical analysis

All statistical analyses were performed using SPSS (IBM™ SPSS 28 for Windows) using the R plugin. Propensity score matching was carried out to exclude confounding variables. Data were analyzed using the Shapiro Wilk *W* normality test, subsequently *T*-test analyses were performed for normally distributed data and the Mann-Whitney *U*-Test for non-parametric equivalents. Statistical significance for all two-tailed tests was set at *P* < 0.05.

## Results

### Operative characteristics

All 148 patients underwent endovascular intervention for aortic arch aneurysm or aortic arch dissection with the RELAY™ Branched endoprosthesis. Technical success was achieved in 147 (99.3%) patients. 17 (11.5%) patients were treated with the single-branched configuration, while 108 (73%) and 23 (15.5%) patients were treated with the double- and triple-branched configurations, respectively. The single (0.67%) mortality in our cohort was subsequent to a technically successful procedure, and the death was not device-related. Operative characteristics are summarized in Table 2. Mean procedural duration was 258 (IQR = 100) min. The modal endovascular duration was 100–150 min (*n* = 95). Endovascular durations are summarized in Table 3.

**Table 2:** Operative characteristics.

Mean procedural time (IQR) (minutes)		258 (100)
Branching number (%)		
	Single	17 (11.5%)
	Double	108 (73%)
	Triple	23 (15.5%)
Technical success (%)		147 (99.3%)
Target vessel cannulation (%)		146 (99.3%)^a^

^a^*Following percentages are calculated out of 147, one case died during the procedure, unrelated to the device*.

**Table 3:** Endovascular duration.

**Duration (min)**	***N* (%)**
50–100	18 (12.2%)
100–150	95 (64.6%)
150–200	24 (16.3%)
200–270	11 (7.5%)

### DS

Over the 24-month follow-up period, in total 6 patients (4.0%) were found to have DS after undergoing TEVAR with the double-branched RELAY™ endoprosthesis. 2 (1.3%) cases of DS were identified within the first 30 days postoperative. A further 2 (1.3%) cases of DS were identified at 6 months postoperative. At 12 months postoperative, 1 (0.7%) patient developed DS. A further single case (0.7%) of DS was identified at 24 months postoperative. The incidence of DS across the 24-month follow-up period is summarized in Table 4.

**Table 4:** Follow-up periods results.

	**30 days**	**6 months**	**12 months**	**24 months**	**Total**
Vessel patency	147 (100%)	134 (91.1%)	124 (84.3%)	118 (80.2%)	–
Reinterventions	8 (5.4%)	6 (4.4%)	5 (4.0%)	5 (4.0%)	24 (16.3%)
Disabling stroke	2 (1.3%)	2 (1.4%)	1 (0.7%)	1 (0.7%)	6 (4.1%)

### Reintervention

Over the 24-month follow-up period, a total of 24 cases of reintervention were recorded. All 24 cases involved patients who had been treated with the double-branched configuration of the RELAY™ Branched endoprosthesis. 8 (5.4%) reinterventions were carried out within the first 30 days postoperative. A further 6 (4.4%) reinterventions were required by 6 months postoperative. An additional 5 (4.0%) reinterventions were recorded by both 12- and 24 months postoperatively. A summary of recorded reinterventions across the 24-month follow-up period is provided in Table 4.

### Target vessel patency

Target vessel patency was maintained in all (147) patients across all three branching configurations at 30 days postoperatively. The 23 (15.6%) patients that were treated with the triple-branched endoprosthesis maintained vessel patency throughout 24 months of follow-up. At 6 months postoperatively, target vessel patency was maintained in 15 (10.2%) and 96 (65.3%) patients that were treated with the single- and double- branched endoprosthesis, respectively. All 16 (10.9%) patients treated with the single-branched endoprosthesis exhibited target vessel patency at 12 months postoperatively, compared to 85 (57.8.%) of those treated with the double-branched configuration. At 24 months postoperatively, 15 (10.2%) and 80 (54.4%) patients treated with the single- or double- branched configurations were noted to have maintained target vessel patency. These findings are summarized in Tables 4, 5.

**Table 5:** Relationship between branching number and vessel patency.

	**Single**	**Double**	**Triple**	**Total**	***P***
30 days	16 (10.9%)	108 (73.4%)	23 (15.6%)	147 (100%)	–
6 months	15 (10.2%)	96 (65.3%)	23 (15.6%)	134 (91.1%)	0.080
12 months	16 (10.9%)	85 (57.8%)	23 (15.6%)	124 (84.3%)	<0.001
24 months	15 (10.2%)	80 (54.4%)	23 (15.6%)	118 (80.2%)	0.001

There was no relationships observed between the mean rank of endovascular duration and incidence of DS across all four follow-up intervals (30 days: *U* = 263, *P* = 0.782; 6 months: *U* = 351, *P* = 0.476; 12 months: *U* = 222, *P* = 0.152; 24 months: *U* = 204, *P* = 0.326). The Mann-Whitney *U* test also revealed a statistically significant relationship between endovascular duration and reintervention rates from 30 days to 12 months postoperative (30 days: *U* = 642, *P* = 0.011; 6 months: *U* = 433, *P* = 0.019; 12 months: *U* = 518, *P* = 0.037) however this relationship was shown to not be statistically significant at the 24-month interval (*U* = 490, *P* = 0.055). Finally, our analysis revealed a relationship between endovascular duration and target vessel patency at the 6- and 24-month intervals (6 months: *U* = 560, *P* = 0.032; 24 months: *U* = 1,283, *P* = 0.035), however this relationship was found to not be significant at the 12-month interval (*U* = 1,072, *P* = 0.056). We note that because target vessel patency remained at 100% across the entire cohort at the 30-day follow-up interval, the Mann-Whitney *U* test was not applied to this sub-group. These findings are summarized in Tables 6–8.

**Table 6:** Endovascular time mean ranks vs. reintervention in each period.

	**Mean ranks (reintervention** = **yes)**	**Mean ranks (reintervention** = **no)**	**Mann-Whitney** ***U***	***P***
30 days	48.63	77.1	642	0.011
6 months	45.36	76.32	433	0.019
12 months	49.67	76.16	518	0.037
24 months	50.59	75.89	490	0.055

**Table 7:** Endovascular time mean ranks vs. disabling strokes in each period.

	**Mean ranks (disabling stroke**= **yes)**	**Mean ranks (disabling stroke**= **no)**	**Mann-Whitney** ***U***	***P***
Post-operative	50.25	76.9	668	0.017
30 days	79.75	73.84	263	0.782
6 months	86.0	73.49	351	0.476
12 months	100.5	73.07	222	0.152
24 months	94.38	73.43	204	0.326

**Table 8:** Endovascular time mean ranks vs. target vessel patency in each period.

	**Mean ranks (vessel patency**= **yes)**	**Mean ranks (vessel patency**= **no)**	**Mann-Whitney** ***U***	***P***
30 days	74	0	-	-
6 months	76.32	50.08	560	0.032
12 months	76.85	58.61	1,072	0.056
24 months	77.63	59.24	1,283	0.035

## Discussion

The endovascular treatment of aortic arch pathologies using the RELAY™ Branched endoprosthesis yields desirable clinical outcomes, both in the short- and intermediate-term. The index procedures included in this series showed a 99.3% (*n* = 147) technical success rate and out of the cohort of 148 patients only one mortality was recorded over the 24-month follow-up period. The incidence of DS was 4.1% (*n* = 6) over 24 months with none treated with a single- or triple-branched RELAY™ endoprostheses. Similarly, none of the patients in these two subgroups required reintervention at any point during the follow-up period. Overall, endovascular arch repair with RELAY™ Branched was associated with a 16.3% (*n* = 24) reintervention rate, and by the 24-month interval, 80.2% (*n* = 118) of patients maintained target vessel patency. All patients (*n* = 23, 15.6%) treated with the triple-branched configuration maintained full target vessel patency throughout follow-up. Furthermore, the data show that the Relay™ Branched system enables rapid deployment of the endoprosthesis at the target site, with the modal endovascular duration in our series being 100–150 min.

The outcomes reported in the present series emphasize that despite the numerous advantages conferred by its minimally-invasive nature, endovascular repair of the aortic arch still carries significant risk of DS, loss of vessel patency, and the need for reintervention. These are complications that adversely impact a patient's quality of life, albeit at a lower rate than that of open surgical repair (17). Indeed, our data suggest that RELAY™ Branched is associated with a more favorable neurological risk profile than that reported by colleagues, whilst also ensuring that clinicians treat these complexities with on-label device use at all times (8, 15, 18).

Tazaki et al., in their analysis of outcomes associated with the Inoue™ triple-branched endoprosthesis, report a combined stroke rate of 40%, while Sato et al. reported a 16.7% (*n* = 6) stroke rate with the Najuta™ fenestrated endograft (16, 18). Czerny et al. observed a combined stroke rate of 20% in their series (8). Additionally, Sato et al. reported that aneurysmal shrinkage post-intervention was observed in only 11 (30.6%) patients, while no change in aneurysmal size was observed in 15 (41.7%) (16). However, increased aortic arch diameter was reported in 27.8% (*n* = 10) of patients in their series (16). In contrast, stable patency of the aortic arch, distal aorta, and the supra-aortic branches was observed in 80.2% (*n* = 11.8) of patients in our study. Iwakoshi and colleagues reported an 83.5% freedom from reintervention rate in their series evaluating the Najuta™ fenestrated endograft (19). This paper shows similar outcomes, with an 83.7% freedom from reintervention rate at 24 months 24 reinterventions occurred during the follow-up period, of which 23 (95.8%) cases were recorded as having taken place in the South Europe region, accounting for 19.6% (*n* = 29) of the total procedures included in the series (Table 9).

**Table 9:** Geographical distribution of reinterventions.

	**West**	**East**	**South**	**North**	**Total**	***P***
30 days	0	0	8	0	8 (5.4%)	<0.001
6 months	1	0	5	0	6 (4.4%)	<0.001
12 months	0	0	5	0	5 (4.0%)	<0.001
24 months	0	0	5	0	5 (4.0%)	<0.001

A significant inverse relationship was seen between the duration of the endovascular procedure and reintervention rates at 30 days, 6 and 12 months. This is suggestive of lower reintervention rates within 1 year post-stenting in those undergoing prolonged endovascular intervention. Though the mechanism behind this relationship remains unclear, it is reasonable to suggest those with complex arch disease require more extensive and prolonged endovascular repair to achieve optimum results.

Following open surgical repair for acute type A aortic dissection, reduced reintervention rates are a well-documented benefit of extensive repair (in comparison to a more conservative approach) (20). Therefore, the statistically observed relationship highlighted may well be an indirect one; the reduced reintervention rate observed up to 12 months may not due to a prolonged procedural duration but a consequence of more extensive aortic repair.

A statistically significant relationship between endovascular duration and target vessel patency at 6- and 24 months postoperative was observed. This finding shows target vessel patency at 6 months is associated with a prolonged endovascular duration and a reduced likelihood for reintervention at 6 months' follow-up. Similarly, prolonged endovascular duration may be the product of more extensive aortic arch repair contributing to improved patency rates of the supra-aortic branches at 6 months.

A treatment option for patients undergoing endovascular aortic arch repair with single- or double-branched endoprostheses is occlusion of the LSA origin, either with extraanatomical revascularization or allowing collateralization to maintain perfusion to the territory supplied by the LSA (21). The data also demonstrate that there does not exist a statistically significant relationship between endovascular duration and the occurrence of perioperative DS at all follow-up intervals. This may perhaps be because perioperative stroke in the context of endovascular aortic arch repair is usually ischemic (as a result of supra-aortic branch occlusion by endoluminal instrumentation or the deployed endoprosthesis) or embolic (due to embolization of particulate matter from the diseased aortic intima during endovascular manipulation of the aorta) (22). Since aortic arch TEVAR is carried out under general anesthetic, without the need for cardiopulmonary bypass, systemic cooling, and circulatory arrest, it is unlikely that procedural duration exerts any direct or independent effect over the pathogenesis of perioperative neurological injury (14). As a result, it could be argued that anesthetics of aortic arch TEVAR may involve no greater risk of perioperative stroke than would be expected for other elective procedures carried out under general anesthetic. In contrast, prolonged procedural duration has traditionally been regarded as a driving factor behind perioperative stroke in the context of open surgical aortic repair, given the need for circulatory arrest and cardiopulmonary bypass.

## Conclusion

RELAY™ Branched TEVAR for the aortic arch represents a promising step forward in on-label endovascular therapy for aortic arch pathology. Though significant advances have been made in this field, the risk of perioperative stroke, loss of vessel patency, and reintervention remain in the fore. Our investigation into the relationship between endovascular duration and the pathogenesis of these complications suggests that prolonged endovascular duration may indirectly correlate with improved target vessel patency and reintervention rates up to 12- and 6 months, respectively. Our analyses revealed that there it is unlikely that endovascular duration affects the incidence of perioperative DS. Further prospective research across different endoprosthetic devices is recommended.

## Data availability statement

The original contributions presented in the study are included in the article/supplementary material, further inquiries can be directed to the corresponding author.

## Ethics statement

Ethical review and approval was not required for the study on human participants in accordance with the local legislation and institutional requirements. Written informed consent for participation was not required for this study in accordance with the national legislation and the institutional requirements.

## Author contributions

ST, AS, and MJ contributed to the drafting of the manuscript. AS was responsible for data analysis. MJ, DB, IW, and MB were responsible for critical review and feedback. All authors contributed to the article and approved the submitted version.

## Funding

DB was a Royal Society Wolfson Research Fellow (#WM170007).

## Conflict of interest

The authors declare that the research was conducted in the absence of any commercial or financial relationships that could be construed as a potential conflict of interest.

## Publisher's note

All claims expressed in this article are solely those of the authors and do not necessarily represent those of their affiliated organizations, or those of the publisher, the editors and the reviewers. Any product that may be evaluated in this article, or claim that may be made by its manufacturer, is not guaranteed or endorsed by the publisher.
